# Insulin and Leptin Signaling Interact in the Mouse Kiss1 Neuron during the Peripubertal Period

**DOI:** 10.1371/journal.pone.0121974

**Published:** 2015-05-06

**Authors:** Xiaoliang Qiu, Hoangha Dao, Mengjie Wang, Amelia Heston, Kaitlyn M. Garcia, Alisha Sangal, Abigail R. Dowling, Latrice D. Faulkner, Scott C. Molitor, Carol F. Elias, Jennifer W. Hill

**Affiliations:** 1 Department of Physiology and Biophysics, Stony Brook University School of Medicine, Stony Brook, New York, United States of America; 2 Department of Bioengineering, University of Toledo, Toledo, Ohio, United States of America; 3 Center for Diabetes and Endocrine Research, Department of Physiology and Pharmacology, University of Toledo College of Medicine, Toledo, Ohio, United States of America; 4 Center for Reproductive Genomics, Cornell University, Ithaca, NY, United States of America; 5 Department of Molecular and Integrative Physiology, University of Michigan, Ann Arbor, MI, United States of America; 6 Department of Obstetrics-Gynecology, University of Toledo College of Medicine, Toledo, Ohio, United States of America; University of Cordoba, SPAIN

## Abstract

Reproduction requires adequate energy stores for parents and offspring to survive. *Kiss1* neurons, which are essential for fertility, have the potential to serve as the central sensors of metabolic factors that signal to the reproductive axis the presence of stored calories. Paradoxically, obesity is often accompanied by infertility. Despite excess circulating levels of insulin and leptin, obese individuals exhibit resistance to both metabolic factors in many neuron types. Thus, resistance to insulin or leptin in *Kiss1* neurons could lead to infertility. Single deletion of the receptors for either insulin or the adipokine leptin from *Kiss1* neurons does not impair adult reproductive dysfunction. However, insulin and leptin signaling pathways may interact in such a way as to obscure their individual functions. We hypothesized that in the presence of genetic or obesity-induced concurrent insulin and leptin resistance, Kiss1 neurons would be unable to maintain reproductive function. We therefore induced a chronic hyperinsulinemic and hyperleptinemic state in mice lacking insulin receptors in Kiss1 neurons through high fat feeding and examined the impact on fertility. In an additional, genetic model, we ablated both leptin and insulin signaling in *Kiss1* neurons (IR/LepR^Kiss^ mice). Counter to our hypothesis, we found that the addition of leptin insensitivity did not alter the reproductive phenotype of IR^Kiss^ mice. We also found that weight gain, body composition, glucose and insulin tolerance were normal in mice of both genders. Nonetheless, leptin and insulin receptor deletion altered pubertal timing as well as LH and FSH levels in mid-puberty in a reciprocal manner. Our results confirm that *Kiss1* neurons do not directly mediate the critical role that insulin and leptin play in reproduction. However, during puberty kisspeptin neurons may experience a critical window of susceptibility to the influence of metabolic factors that can modify the onset of fertility.

## Introduction

Obesity and hyperinsulinemia are associated with infertility [1]. Hypogonadotrophic hypogonadism, a state in which the release of hormones from all levels of the reproductive axis is reduced, is the most common reproductive consequence of type 2 diabetes and obesity [2, 3]. In obese women, LH [4, 5], LH pulse amplitude [6], follicle-stimulating hormone (FSH) [2, 4, 5], and progesterone [4–6] are significantly lower than in normal weight women. LH pulse amplitude is also lower in obese men [7]. Similarly, more than 25% of men with type 2 diabetes have hypothalamic hypogonadism [8]. While obesity and insulin resistance are each independently associated with lower plasma testosterone levels [9, 10], obese men with T2DM appear to be at particularly high risk [11, 12].

These metabolic factors have an impact on GnRH release. Obesity is associated with high levels of leptin production by adipocytes. Circulating leptin is required for maintaining GnRH release [13–15]. Interestingly, GnRH neurons themselves do not sense leptin, implicating upstream neurons in that role [16–18]. Insulin signaling also plays a role in central reproductive control. Female mice with disruption of the insulin receptor in the brain have lower LH levels and subfertility [19]. Moreover, the hypogonadism of sheep or rats lacking pancreatic insulin secretion can frequently [20–24] but not always [25] be ameliorated by central administration of insulin. Therefore, the preponderance of evidence suggests that central insulin signaling promotes GnRH release. While hyperinsulinemia and hyperleptinemia would therefore be expected to increase GnRH production, it does not. While the cause of low GnRH release in obese patients is unclear, insulin and leptin resistance develops in some neuronal circuits during obesity in a manner similar to insulin resistance in peripheral tissues [26–30]. Evidence supporting leptin resistance in neurons controlling GnRH in obese animals comes from hyperleptinemic mutant mice, which develop hypogonadotropic hypogonadism during middle age [31]. However, whether insulin resistance develops in neurons controlling GnRH release is not yet known.

It has been hypothesized that obesity acts via *Kiss1* neurons to cause secondary hypogonadism [32]. Kisspeptin (a product of the *Kiss1* gene) is a key hypothalamic neuropeptide that stimulates GnRH release in mice and humans [33–37]. Body weight, nutrition, metabolism, and hormone levels may influence the activity of *Kiss1* neurons [38, 39]. Tissue specific knockout studies indicate that absence of the insulin or leptin receptor in kisspeptin neurons does not alter LH levels in adult lean mice [40–43]. The function of these neurons in a chronic state of hyperinsulinemia and hyperleptinemia, however, has not been tested. Previous studies have demonstrated that intracellular signaling pathways activated by insulin and leptin overlap and may thereby exert similar effects [44–47], but it is unclear whether insulin and leptin play a cooperative role in maintaining *Kiss1* neuron function. To determine the role of insulin receptors in the reproductive neuroendocrine dysfunction associated with obesity, we used a mouse model of insulin receptor (IR) deletion in kisspeptin neurons [42] and induced hyperinsulinemia with high fat feeding. Then, using transgenic KO mice with *Kiss1*-specific deletion of both the insulin and leptin receptor, we investigated the effects of simultaneously impaired leptin and insulin signaling in *Kiss1* neurons on fertility and energy metabolism.

## Materials and Methods

### Animals and genotyping

To generate mice with the IR specifically deleted in *Kiss1* neurons (IR^Kiss^ mice), *Kiss1*-Cre mice [48] were crossed with insulin receptor floxed mice [19] and bred to homozygosity for the floxed allele only. The IR^flox/flox^ mice were designed with loxP sites flanking exon 4. Excision of exon 4 in the presence of Cre recombinase results in a frameshift mutation and produces a premature stop codon. Littermates lacking Cre expression served as controls (IR^flox/flox^). All mice were on a mixed C57BL/6J-129S6/SvEv background.

To generate mice with the IR and leptin receptor (LepR) specifically deleted in *Kiss1* neurons (IR/LepR^Kiss^ mice), *Kiss1*-Cre mice [48] were first crossed with insulin receptor floxed mice [19] and bred to homozygosity for the floxed allele only. These mice were then crossed with mice carrying a loxp flanked LepR to produce IR/LepR^Kiss^ mice. Exon 17 of LepR was flanked by loxP sites, containing the Box 1 motifs crucial for leptin signal transduction through JAK2 and STAT3 pathways [49]. Cre-mediated recombination produces a LepR that lacks exon 17 and has a truncated missense exon 18, which has previously been reported to recapitulate phenotypes found in the LepR^db/db1J^ mutation [49–51]. All mice were on a mixed C57BL/6J-129S6/SvEv background.

Mice were housed in the University of Toledo College of Medicine animal facility at 22–24°C on a 12h light/12h dark cycle and were fed standard rodent chow 2016 Teklad Global 16% Protein Rodent Diet (12% fat by calories, Harlan Laboratories, Indianapolis, Indiana). On postnatal day (PND) 22, mice were weaned. Mice were used only if litter size was within 5 to 10 pups to prevent birth size effects on body weight. At the end of the study, all animals were sacrificed by CO_2_ asphyxiation or by cardiac puncture under 2% isoflurane anesthesia to draw blood. All procedures were reviewed and approved by University of Toledo College of Medicine Animal Care and Use Committee. Mice were genotyped as previously described [46, 48] (Table 1). Additional genotyping was carried out by Transnetyx, Inc. (Cordova, Tennessee) using a real-time PCR-based approach.

**Table 1:** Genotyping primer sequences.

	Forward primer	Reverse primer
IR-loxP	gatgtgcaccccatgtctg	ctgaatagctgagaccacag
LepR- loxP	caggcttgagaacatgaacacaacaac	aatgaaaaagttgttttgggacga
Kiss-Cre	tgcgaacctcatcactcgttgcat	gctctggtgaagtacgaactctga
IR-delta	gggtaggaaacaggatgg	ctgaatagctgagaccacag
LepR-delta	gtctggaccgaaggtgttagtgag	cagtaagcagaaagagagatagatgtgttg

### Electrophysiology

*Kiss1*-cre mice were crossed with an enhanced green fluorescent protein (EGFP) reporter strain (B6;129-Gt(ROSA)26Sortm2Sho/J; Jackson Laboratory) to create mice that produce EGFP in cre-expressing neurons. Male offspring 6–8 weeks in age were anesthetized with ketamine (80 mg/kg) and xylazine (20 mg/kg) and decapitated. The forebrain was rapidly removed into ice-cold artificial cerebrospinal fluid (ACSF) containing (in mM) 127.3 NaCl, 1.3 KCl, 1.3 KH2PO4, 21.4 NaHCO3, 5 glucose, 2.5 CaCl2 and 1.3 MgSO4. Coronal slices 250–300 μm thick containing hypothalamus were obtained using a vibrating microtome (Vibratome, St. Louis MO) and placed in an incubation beaker with room-temperature ACSF continuously perfused with 95% O2–5% CO2. Following dissection, the incubation temperature was slowly raised to the recording temperature of 32°C. Slices remained in incubation at least 1 hour before being transferred to the recording chamber and were superfused with the 0.5–2 ml/min ACSF maintained at 32°C and continuously perfused with 95% O2–5% CO2 throughout all recordings.

Viable arcuate or periventricular neurons were visualized with infrared video microscopy on an Olympus BX51W1 fixed-stage upright microscope with a 40X, 0.8 N.A. water immersion objective. Cre-expressing *Kiss1* neurons that produce EGFP using epifluorescence provided by a shuttered mercury arc lamp (HBO 100 W, Olympus) and a fluorescein filter set having a 465–495 nm excitation filter, a 505 nm dichroic, and a 515–555 nm emission filter (Chroma Technology, Rockingham VT). Whole-cell recordings were obtained using a Multiclamp 700A amplifier (Axon Instruments, Union City CA) in current clamp mode, filtered at 10 kHz and digitized at 50 kHz with a 12-bit A/D converter (National Instruments, Austin TX) using customized software developed under MATLAB (Mathworks, Natick MA). The pipette solution contained 120 K gluconate, 10 KCl, 0.3 CaCl2, 1 MgCl2, 5 MgATP, 10 HEPES, 0.3 NaGTP, 1.0 EGTA, pH 7.3 with 3–5 mM KOH.

Recording pipettes were pulled from borosilicate glass capillary tubing (KG33, Garner Glass, Claremont CA) and have a final resistance of 8–12 MΩ. Recordings were deemed acceptable if Vm < -50 mV (corrected for-10 mV junction potential) and access resistance < 25 MΩ immediately after the whole-cell configuration was obtained. If needed, small amounts of hyperpolarizing holding current (typically less than 200 pA) were applied to prevent spontaneous action potentials (APs). Insulin was applied using 1–5 MΩ pressure pipettes filled with 200 nM insulin in ACSF. A sequence of 1, 2, 3 or 4 x 20 ms pressure pulses was applied during traces obtained every 10 sec and interspersed with control traces in which no pressure pulses were applied. Responses were averaged across 6–12 traces obtained from an individual cell and statistically significant changes in membrane potential produced by 1–4 insulin pulses relative to control traces without insulin application were identified by one-way analysis of variance.

### Diet-induced obesity (DIO) Studies

One group of IR^Kiss^ mice and littermate controls was placed on high fat diet with 60% kcal from fat (5.24kcal/g; Research Diets, Inc.; New Brunswick, NJ) at 8 weeks old for 5 months. During the feeding, the mice were measured for body weight every week. After 3 months of high fat diet treatment, NMR was performed to assess body composition. Fasting serum was obtained for insulin and leptin measurements, and non-fasting serum was obtained for LH, and FSH measurements. At the age of 4–5 months, mice were paired with established breeders for at least 1 month. If a mouse did not produce a new litter after 2 months of breeding, it was considered to be sterile. Date of birth of new litters and litter size were recorded if a litter was produced. A second group of IR^Kiss^ mice and littermate controls received high fat diet from the day of weaning (PND 21). Body weight was measured every 3 days until 8 weeks old. Vaginal opening and cytology were checked every day until 8 weeks old.

### Puberty and reproductive phenotype assessment

Balanopreputial separation was checked daily from weaning by manually retracting the prepuce with gentle pressure [52]. Singly housed female mice were checked daily for vaginal opening after weaning at 3 weeks of age. Vaginal lavages from female mice were collected from the day of vaginal opening for at least 3 weeks. Stages were assessed based on vaginal cytology [53, 54]; predominant cornified epithelium indicated the estrous stage, predominant nucleated cells indicated the proestrus stage, and predominant leukocytes indicated the diestrus stage. At the age of 33 days, each male mouse was paired with 1 wild-type female mouse of proven fertility for 4 weeks or until the female mouse was obviously pregnant. Then the paired mice were separated, and the delivery date was recorded. The age of sexual maturation was estimated from the birth of the first litter minus average pregnancy duration for mice (20 days). At 4 to 6 months of age, animals were again paired with wild-type adult breeders to collect additional data on litter size and intervals between litters.

### Metabolic phenotype assessment

Body weight was measured weekly in a single-occupant cage with ALPHA-dri bedding. Body composition in 4 month old mice was assessed by nuclear magnetic resonance (minispec mq7.5, Bruker Optics, Billerica, Massachusetts) to determine the percentage of fat mass, as previously described [55]. Glucose tolerance tests (GTT) and insulin tolerance tests (ITT) were done as previously described[46]. Briefly, following overnight fast, tail blood glucose was measured using a mouse-specific glucometer (AlphaTRAK, Abbott, Abbott Park, Illinois) before and 15, 30, 60, 90 and 120 min after dextrose (2g/kg, i.p.) injection. For ITT, following a 4 hour fast, mice were injected with recombinant insulin (0.75U/kg, i.p.). Tail blood glucose was measured again at specified time points. Food intake was measured every week until mice were 4 months old.

### Quantitative real-time PCR

Mice were decapitated after isoflurane anesthesia and brains and other tissues were removed. Total RNA were extracted from dissected tissues by RNeasy Lipid Tissue Mini Kit (Qiagen) and single-strand cDNA was synthesized by High-Capacity cDNA Reverse Transcription kit (Applied Biosystems) using random hexamers as primers. 10μM cDNA template was used in a 25μl system in 96-well plates by SYBR green qPCR SuperMix/ROX (Smart Bioscience). Each sample was analyzed in triplicate to measure gene expression level. The reactions were run in ABI PRISM 7000 sequence detection system (PE Applied Biosystems) and analyzed using the comparative Ct method (2^-ΔΔCt^) with GAPDH as the normalizer.

### Hormone assays

Submandibular blood was collected at 10:00–11:00AM to detect basal LH and FSH levels using the rat pituitary panel (Millipore), performed by the University of Virginia Center for Research in Reproduction. We chose this time point to avoid the LH surge in randomly cycling mice. The assay for LH had sensitivity of detection of 3.28 pg/ml. The intra-assay and inter-assay coefficients of variance (CV) were 6.9% and 17.2%, respectively. For FSH the lower limit of detection was 7.62pg/ml, with intraassay and interassay CV 6.7% and 16.9%, respectively. Serum estradiol was measured by ELISA (Calbiotech, Spring Valley, California) with sensitivity <3pg/ml, and intra-assay CV 3.1% and interassay CV 9.9%. Serum testosterone was also measured by ELISA (Calbiotech). Serum collected after an overnight fast was used for measurement of insulin and leptin (Crystal Chem Inc. Downers Grove, Illinois). The leptin ELISA has a sensitivity range of 0.2 to 12.8ng/ml, with both intraassay and interassay CV≤10%. The insulin ELISA kit had a sensitivity range of 50–3200pg/ml, with both intraassay and interassay CV≤10%.

### Perfusion and immunohistochemistry

Adult male mice and female mice at diestrus at the age of 3–6 months were deeply anesthetized by ketamine and xylazine (100 mg/kg and10 mg/kg, respectively). After briefly perfusing with a saline rinse, mice were perfused transcardially with 10% formalin for 10 minutes and the brain was removed. The brain was post-fixed in 10% formalin at 4°C overnight, and then immersed in 20% sucrose at 4°C for 48 hours. 25μm sections were cut with a sliding microtome into 5 equal serials. Sections were treated with 3% hydrogen peroxide for 30 minutes to quench endogenous peroxidase activity. After rinsing in phosphate-buffered solution (PBS), sections were blocked 2h in PBS-azide-T (PBS-azide; Triton X; 3% normal donkey serum). Then, samples were incubated for at least 48 h at 4°C in PBS-azide-T containing rabbit anti-kisspeptin IgG (1:1000, Millipore), which has been tested for specificity [46, 56]. After several washes in PBS, sections were incubated in PBS-T (Triton X, 3% donkey serum) containing biotinylated anti-rabbit IgG (1:1000, Vector Laboratories, Burlingame, California), followed by incubation in ABC reagent (Vector Laboratories) for 60 minutes at room temperature. Sections were washed and immunoreactivity was visualized by 0.6mg/ml diaminobenzidine hydrochloride (Sigma) in PBS with hydrogen peroxide. Finally, sections were washed, mounted on slides, dried overnight, dehydrated, cleared and coverslipped. *Kiss1*-immunoreactive neurons in the AVPV/PeN and ARC were quantified as previously described [56, 57].

### Histology

Ovaries and testes were collected from mice and fixed in 10% formalin overnight. Tissues were embedded in paraffin and cut into 5–8μm sections by the University of Toledo Pathology Department. Sections were stained by hematoxylin and eosin.

### Western blotting

Adult mice were sacrificed, and the hypothalamus, liver, muscle, visceral adipose tissues, and gonads were harvested. Tissues were snap frozen in liquid nitrogen and stored in -80°C until homogenized in radioimmunoprecipitation assay lysis buffer (Millipore, Billerica, Massachusetts) supplemented with protease inhibitor and phosphatase inhibitor (Thermo Scientific, Waltham, Massachusetts). After centrifugation, supernatant protein concentrations were determined by BCA protein assay (Thermo Scientific). 30μg or 50 μg of denatured samples were subjected to SDS-PAGE electrophoresis and western blotting using IR β subunit (1:1000, Santa Cruz Biotechnology Inc. Santa Cruz, California). β-actin (1:1000, Sigma, St Louis, Missouri) or α-tubulin (1:1000, Cell Signaling, Danvers, Massachusetts) was used as a loading control.

### Statistical analysis

Data are presented as the mean ± SEM. Two-tailed unpaired t testing served as the main statistical method. Mann-Whitney U test was used if the data did not assume a normal distribution. One-way analysis of variance (ANOVA) was performed to compare three independent groups, followed by Sidak's multiple comparison test. Paired t testing was employed in the case of single repeated measures. For body weight, GTT and ITT, repeated measures ANOVAs were used to compare changes over time between two genotypes. *P*<0.05 was considered to be statistically significant.

## Results

### Electrophysiological response to insulin

**Fig 1:**
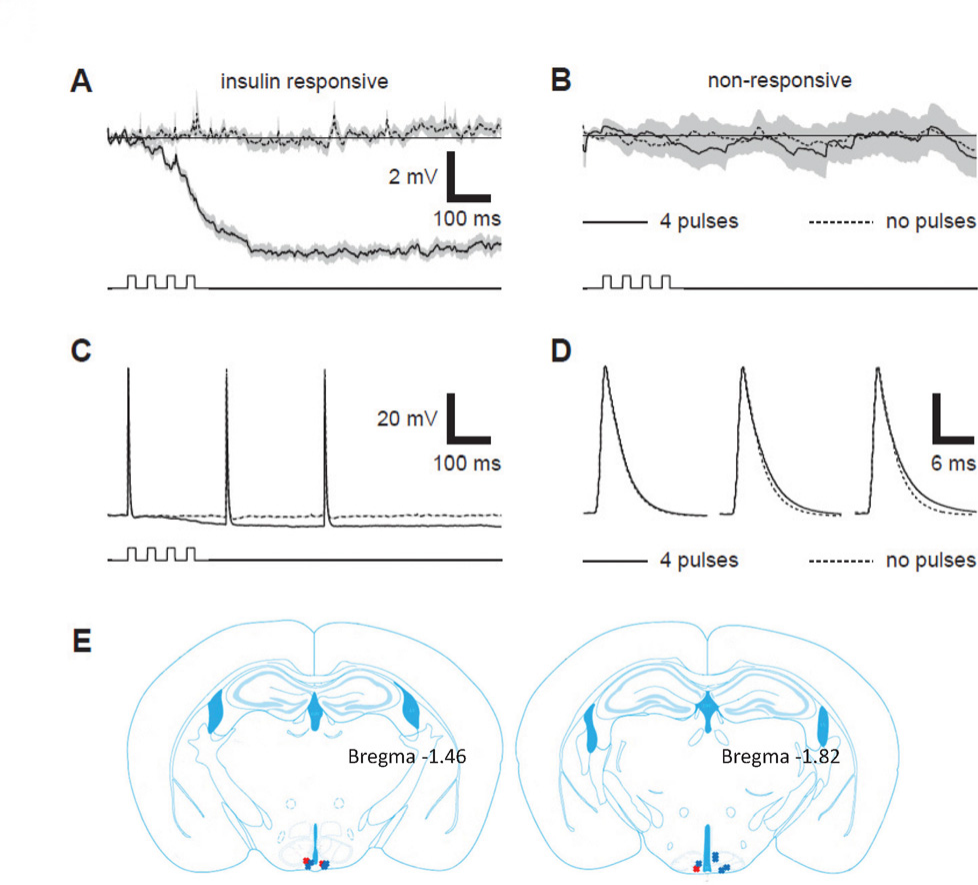
Electrophysiological response of *Kiss1* neurons to insulin. A: Averaged membrane potential (Vm) recordings from an insulin-responsive *Kiss1* neuron elicited with (solid line) and without (dashed line) a 4 x 20 ms pressure application of 200 nM insulin in ACSF. Gray shading represents the mean ± SEM from traces averaged across 11 repetitions obtained every 10 sec. B: same as A with recordings averaged across 11 repetitions from a *Kiss1* neuron that did not respond to pressure pulses of 200 nM insulin. Time course of pressure pulses is shown below Vm traces in A—B. C: recordings from same neuron in A in which APs were elicited by 0.2 ms depolarizing current pulses during (solid line) and without (dashed line) 4 x 20 ms pressure application of 200 nM insulin in ACSF. D: APs from C displayed on magnified time scale and aligned at peak to identify whether any changes occurred in AP time course. E: Locations of recordings from EGFP positive *Kiss1* neurons. Red Xs represent insulin-responsive neurons, and the blue Xs represent insulin nonresponsive neurons. Modified from *Mouse Brain in Stereotaxic Coordinates*, *3* ^*rd*^ *Edition* by Franklin and Paxinos (used with permission).

We previously reported 22% of *Kiss1* neurons in the ARC and 5% of *Kiss1* neurons in the AVPV expressed IR [42]. To determine if these neurons are in fact insulin-responsive, we first examined the responsiveness of *Kiss1* neurons to insulin in mice that produce EGFP in cre-expressing neurons. Electrophysiological responses during current clamp recordings showed hyperpolarizing responses to pressure application of insulin in 26.6% (4 out of 15) of *Kiss1* neurons from 9 animals (Table 2).The majority of responsive neurons were located in the ARC (Fig. 1E). In responsive neurons, sequential pressure pulses of 200 nM insulin in ACSF elicited inhibitory postsynaptic potentials (IPSPs) observed in a dose-dependent manner (Fig. 1A). Other *Kiss1* neurons were not responsive to insulin (Fig. 1B), nor were any *Kiss1* neurons responsive to pressure application of ACSF without insulin (not shown).

**Table 2:** Membrane potential changes in Kiss1 neurons in response to insulin.

cell	IPSP mean ± S.D. relative to no insulin pulses (mV)					N	P
	no insulin pulses	1 insulin pulse	2 insulin pulses	3 insulin pulses	4 insulin pulses		
14jun13b	0.00 ± 0.81	-1.84 ± 0.90	-3.00 ± 1.04	-3.26 ± 1.44	-4.35 ± 0.83	11	0.000
18jun13c	0.00 ± 2.00	-0.81 ± 1.49	-1.21 ± 2.96	-1.95 ± 2.51	-2.98 ± 3.47	7	0.001
18jun13e	0.00 ± 1.31	-0.17 ± 1.76	-1.08 ± 1.68	-0.46 ± 1.67	-1.06 ± 1.45	12	0.015
24may14e	0.00 ± 1.43	0.10 ± 1.50	-1.02 ± 1.80	-0.49 ± 1.02	-1.05 ± 1.28	11	0.031
24sep13e	0.00 ± 3.16	2.29 ± 3.04	-0.59 ± 3.64	1.19 ± 2.56	-0.72 ± 3.80	6	0.305
25sep13j	0.00 ± 2.68	0.11 ± 2.54	-2.33 ± 2.75	0.14 ± 1.55	-0.45 ± 3.96	9	0.295
23may14a	0.00 ± 1.23	0.20 ± 1.65	-0.72 ± 1.12	-0.30 ± 1.85	-0.91 ± 1.35	9	0.152
29may14c	0.00 ± 1.04	0.58 ± 0.76	-0.32 ± 0.60	-0.42 ± 0.92	0.06 ± 1.04	11	0.196
29may14d	0.00 ± 2.41	0.15 ± 1.74	0.82 ± 3.31	0.40 ± 2.75	-1.24 ± 2.92	7	0.610
29may14g	0.00 ± 1.87	0.53 ± 1.66	0.69 ± 1.88	-0.71 ± 2.09	0.24 ± 2.09	10	0.454
23jul14a	0.00 ± 2.44	-0.79 ± 1.98	-0.21 ± 1.88	-0.47 ± 2.17	0.20 ± 2.60	8	0.893
23jul14d	0.00 ± 2.17	0.17 ± 2.78	-0.08 ± 1.63	1.08 ± 1.47	1.37 ± 1.20	11	0.159
23jul14e	0.00 ± 2.50	0.89 ± 3.37	0.10 ± 3.79	-0.99 ± 2.40	-0.66 ± 2.44	8	0.725
24jul14a	0.00 ± 2.08	-0.51 ± 2.78	-0.24 ± 2.05	1.10 ± 1.79	-0.17 ± 2.52	8	0.603
24jul14b	0.00 ± 1.37	-0.41 ± 2.14	0.29 ± 1.19	-0.14 ± 1.44	0.09 ± 1.45	11	0.826

Average membrane potential (Vm) changes from 15 Kiss1 neurons in hypothalamic slices obtained from 9 animals in response to pressure application of 1, 2, 3 or 4 x 20 ms pulses of 200 nM insulin. Insulin-induced Vm changes are relative to the average Vm observed during control traces without insulin application. Averages were obtained from 6–12 repetitions obtained every 10 sec. Statistically significant Vm changes produced by 1–4 insulin pulses relative to control traces without insulin application were identified by one-way ANOVA. Significant responses to insulin application were observed in the first 4 Kiss1 neurons obtained from 3 animals (P < 0.05); remaining 11 Kiss1 neurons from 6 animals did not show any significant responses (P > 0.05).

In addition to traces showing changes in resting Vm in response to pressure application of insulin (e.g. Fig. 1A and B), APs were elicited by brief (0.2 ms) depolarizing current pulses during pressure application of insulin to examine the effects of insulin on voltage-dependent responses. Outside of the superposition of APs upon insulin-evoked IPSPs (Fig. 1C), no substantial changes to the magnitude of APs were observed in any insulin-responsive neurons (Fig. 1D). APs elicited during the insulin-dependent IPSP appeared to repolarize more slowly (Fig. 1D, solid lines), which could be attributed to the reduction in voltage gated K^+^ channel activity at the more hyperpolarized Vm produced by the IPSP.

### Impact of high-fat feeding on body weight and fertility of IR^Kiss^ mice

**Fig 2:**
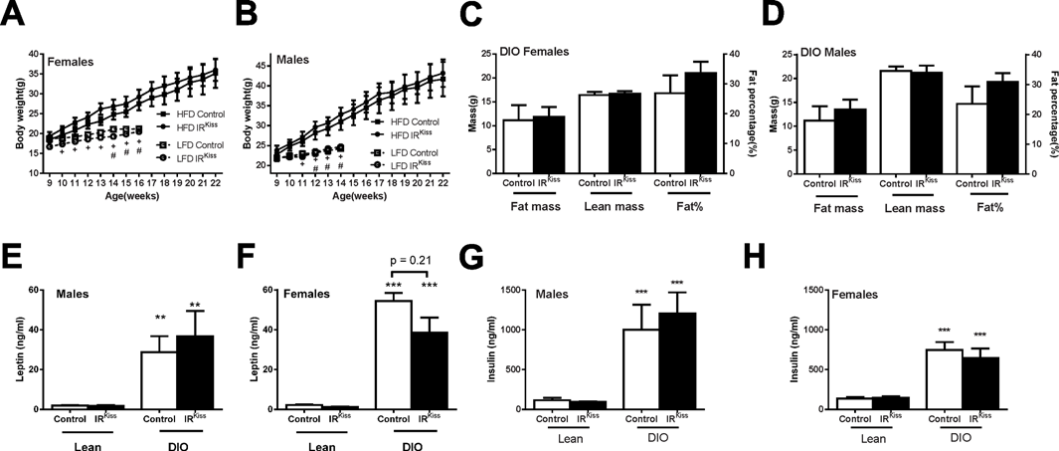
Metabolic parameters in IR^Kiss^ mice after HFD treatment. (A) Female body weight growth curve after HFD (n = 8, 8). Body weights of a low-fat diet cohort included for comparison, + indicates control low and high fat diet groups differ significantly, # indicates IR^Kiss^ low and high fat diet groups differ significantly, * indicates two HFD groups differ significantly. (B) Male body weight growth curve after HFD (Control, n = 7; IR^Kiss^, n = 5). Body weights of a low-fat diet cohort included for comparison. + indicates control low and high fat diet groups differ significantly, # indicates IR^Kiss^ low and high fat diet groups differ significantly. (C) Female body composition measured by NMR 3 months after HFD (n = 8, 8). (D) Male body composition measured by NMR 3 months after HFD (n = 7, 5). (E) Serum leptin levels in lean males (n = 16, 8) and males after 3 months of exposure to HFD (n = 7, 4) (F) Serum leptin levels in lean females (n = 7, 7) and females after 3 months of exposure to HFD (n = 4, 9) (G) Serum insulin levels in lean males (n = 11, 10) and males after 3 months of exposure to HFD (n = 6, 5) (H) Serum insulin levels in lean females (n = 9, 10) and females after 3 months of exposure to HFD (n = 3, 9).

While insulin sensing by *Kiss1* expressing cells has been reported to have minor effects on puberty and serum insulin levels [42, 43], its overall physiological relevance remains unclear. To test whether *Kiss1* insulin sensitivity has heightened importance in an obese, hyperinsulinemic milieu, we treated IR^Kiss^ mice with high fat diet in both peripubertal period and adulthood. In male and female mice treated with high-fat diet (HFD) from 8 weeks old, obesity, hyperinsulinemia and hyperleptinemia developed in control and IR^Kiss^ mice (Fig. 2A-H). However, no difference was detected in these parameters between control and IR^Kiss^ mice. This finding suggests that insulin secretion is normal in this model and that insulin signaling in *Kiss1* neurons does not contribute to the development of obesity.

**Fig 3:**
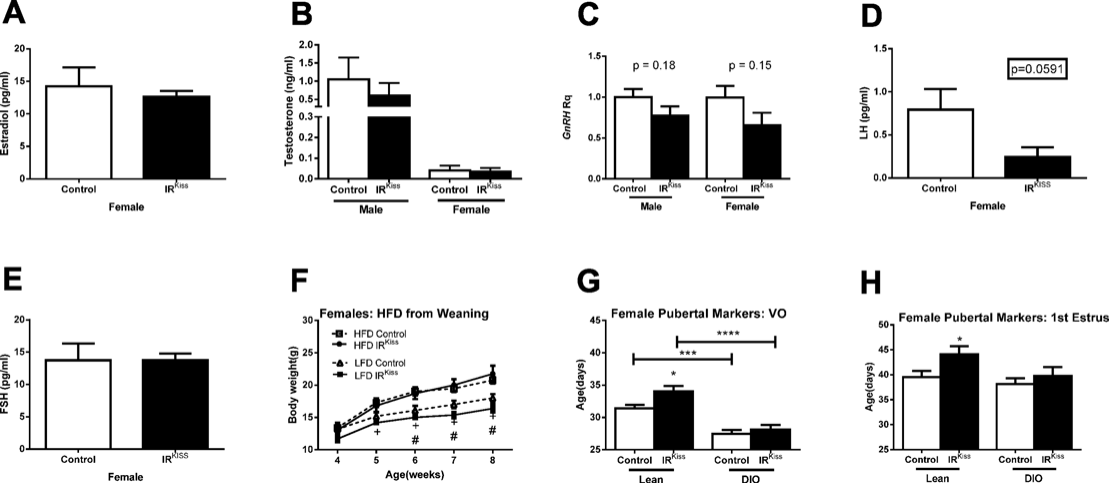
Reproductive axis function in IR^Kiss^ mice after HFD treatment. (A) Serum estradiol in females (n = 3, 6) after 3 months on HFD in adulthood. (B) Serum testosterone levels in males (n = 4, 5) and females (n = 4, 7) after 3 months on HFD in adulthood. (C) GnRH gene expression after 4 months on HFD in adulthood in males (n = 6, 4) and females (n = 5, 4). (D) Serum LH levels in females (n = 13, 6) after 3 months on HFD in adulthood. (E) Serum testosterone levels in females (n = 13, 6) after 3 months on HFD in adulthood. (F) Body weight growth curve in females placed on HFD at weaning (n = 4, 4). + indicates control low and high fat diet groups differ significantly, # indicates IR^Kiss^ low and high fat diet groups differ significantly. (G) Age of vaginal opening and first estrus in females placed on HFD at weaning (n = 11, 10). (H) Age of vaginal opening and first estrus in females placed on HFD at weaning (n = 11, 10).

We also examined the impact of obesity on the fertility of these mice. After 3 months HFD treatment, more than half of female mice had ceased to exhibit estrous cycles (defined as constant estrus or diestrus vaginal cytology of 10 days or more) in both groups (Table 3). After 4 months HFD treatment, 60–70% female mice had stopped cycling regardless of their genotypes (not shown) and failed to become pregnant when paired with a fertile male (Table 4). However, most male mice in both groups retained their fertility (Table 4). Since in some reports, obesity can increase testosterone levels in female mice [58], we examined its levels in both sexes. Control and IR^Kiss^ mice displayed similar estradiol and testosterone levels (Fig. 3A and 3B). Likewise, GnRH expression did not differ significantly in males and females of either genotype, although a trend toward lower GnRH expression was seen in IR^Kiss^ mice (Fig. 3C). Control and IR^Kiss^ mice displayed similar LH and FSH levels (Fig. 3D and 3E). Finally, given our previous finding that insulin signaling in kisspeptin neurons contributes to the timing of puberty in lean mice, we examined whether exposure to HFD from the age of weaning would exacerbate this effect. HFD advanced the age of vaginal opening in all mice, although the timing of first estrus was unaffected. As previously reported, IR^Kiss^ mice exhibited a significant delay in the timing of vaginal opening and first estrus. However, the timing of vaginal opening and first estrus was similar in both groups following high-fat feeding (Fig. 3, F-H). Therefore, (emerging) obesity can advance puberty in females without insulin sensing by kisspeptin neurons. It remains to be determined whether a stronger challenge, for example with maternal high-fat feeding, would show similar results.

**Table 3:** Estrous cycle after 3 months HFD treatment.

	Control	IR^Kiss^
Normal cycle	4	2
No cycle	4	5

**Table 4:** Fertility after 4 months HFD treatment.

		Control	IR^Kiss^
Female	Fertile	3	4
	Sterile	5	6
Male	Fertile	6	5
	Sterile	1	0

### Kisspeptin immunoreactivity in IR/LepR^Kiss^ mice

**Fig 4:**
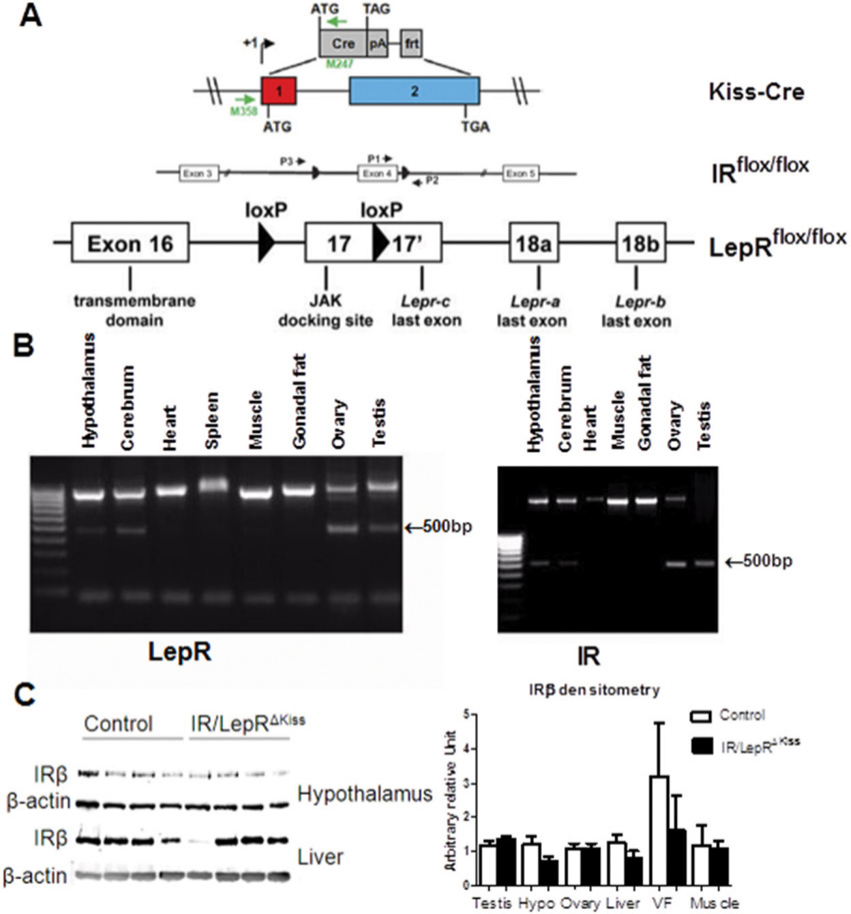
Generation of IR/LepR^ΔKiss^ mice. (A) Construct in making IR/LepR^ΔKiss^ mice. Adapted from previous publications [48, 50, 81]. (B) PCR of DNA from different tissues. The exercised LepR and IR both appear as a 500bp band, whereas unexercised LepR shown as 1kb band and unexercised IR as a 2.2kb band. (C) Representative IRβ expression in different tissues and densitometry.

**Fig 5:**
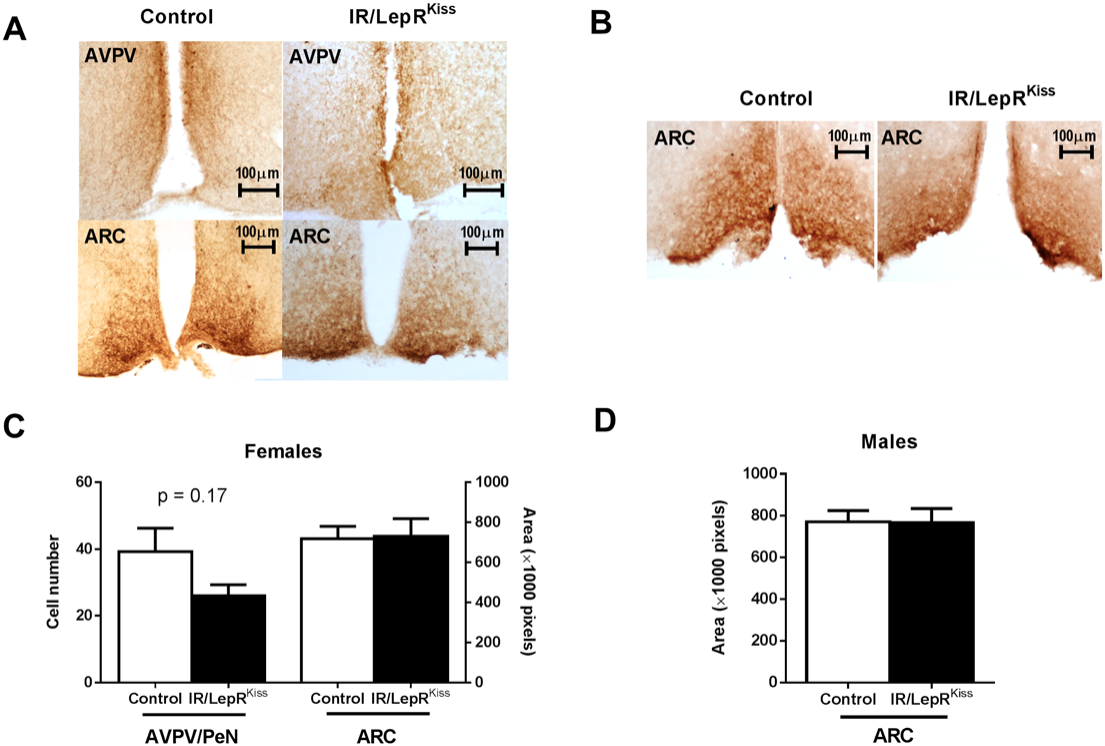
Kisspeptin cell number and process density in IR/LepR^Kiss^ females. (A) Female representative *Kiss1* immunostaining: Upper left square = AVPV/PeN of control mouse, lower left square = ARC of control mouse, upper right square = AVPV/PeN of IR/LepR^Kiss^ mouse, lower right square = ARC of IR/LepR^Kiss^ mouse. Left square = ARC of control mouse, right square = ARC of IR/LepR^Kiss^ mouse. (B) Male representative *Kiss1* immunostaining (C) Quantification of *Kiss1* cell number in the AVPV/PeN (n = 2, n = 4 mice) and *Kiss1* immunoreactive area in the ARC (n = 4, 4) of adult females. (D) Quantification of *Kiss1* immunoreactive area in the ARC in adult males (n = 3, 3). AVPV/PeN, anteroventral periventricular and anterior periventricular nuclei; ARC, arcuate nucleus.

These foregoing results suggest that in an obese state, insulin signaling does not alter the actions of kisspeptin neurons. However, it is possible that leptin signaling could obscure the effects of insulin signaling in some *Kiss1* neurons, given their partially overlapping downstream signaling cascades [44–47]. We therefore generated mice with the IR and LepR specifically deleted in *Kiss1* neurons. To do so, we crossed LepR^flox/flox^ mice with IR^Kiss^ mice carrying the Cre recombinase gene driven by the *Kiss1* promoter as previously described [42, 59](Fig. 4A). To verify that floxed IR and LepR gene was excised in *Kiss1* neurons, PCR was performed on DNA from different tissues. As expected, a 500bp band indicating IR and LepR gene deletion was produced from the hypothalamus and other tissues expressing *Kiss1*, including cerebrum, ovary and testis (Fig. 4B) [48, 59, 60]. IR protein levels were similar between wildtype and targeted-knockout liver, muscle, visceral fat tissue, testis and ovary (Fig. 4C). Consistent with restriction of IR inactivation to a defined subpopulation of hypothalamic neurons, western blot analysis revealed no alteration of IR expression in hypothalamus (Fig. 4C) [61]. Thus, insulin sensing appears to be intact in the majority of cells in the ovary and other tissues. Since leptin and insulin may alter neuronal development [62–66], we examined the number of *Kiss1* neurons and fibers in IR/LepR^Kiss^ mice. Normal patterns of kisspeptin immunoreactivity were seen in both AVPV and ARC in adult mice in both sexes (Fig. 5A, 5B). We counted the *Kiss1* neuron number in the AVPV/PeN and measured the *Kiss1* immunoreactive areas in the ARC from adult mice. There was no difference in either parameter after quantification (Fig. 5C, 5D). Thus, leptin and insulin signaling are not required for adult *Kiss1* gene expression or neuron survival.

### Puberty in IR/LepR^Kiss^ mice

**Fig 6:**
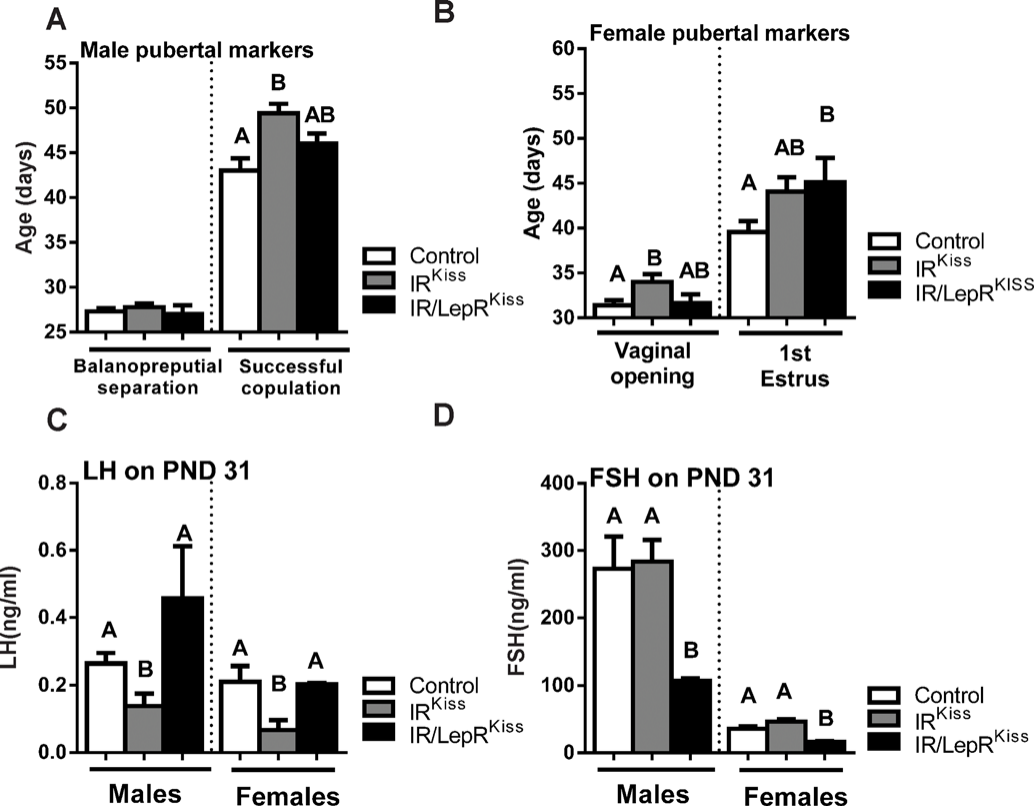
Puberty in IR/LepR^Kiss^ mice. (A) Balanopreputial separation age in male control (n = 12), IR^Kiss^ (n = 10) and IR/LepR^Kiss^ (n = 3) mice. Estimated age of sexual maturation calculated by subtraction of 20 days from the time required for delivery of a litter sired by male control (n = 8), IR^Kiss^ (n = 10) and IR/LepR^Kiss^ (n = 4) mice. (B) Vaginal opening and first estrus was evaluated in female control (n = 9–12), IR^Kiss^ (n = 10) and IR/LepR^Kiss^ (n = 7–8) mice. (C) LH serum levels on postnatal day 31 in male control (n = 7) and IR/LepR^Kiss^ (n = 7) mice and female control (n = 10) and IR/LepR^Kiss^ (n = 6) mice. (D) FSH serum levels on postnatal day 31 in male control (n = 7) and IR/LepR^Kiss^ (n = 7) mice and in female control (n = 10) and IR/LepR^Kiss^ (n = 6) mice.

Since we have demonstrated a delay in puberty compared to wild-type controls after deleting IRs from *Kiss1* neurons, we tested whether the additional LepR deletion would modify the pubertal phenotype of the mice. In males, we saw no alteration in the age of balanopreputial separation among the three groups, although we may have failed to detect a significant difference due to low statistical power. The age at which males were able to impregnate a female was delayed by approximately 6 days in the IR^Kiss^ mice, while the deletion of LepRs from Kiss1 neurons resulted in an intermediate phenotype (Fig. 6A). Interestingly, the LH levels of males on postnatal day 31 was significantly decreased in IR^Kiss^ males and restored in IR/LepR^Kiss^ males (Fig. 6C). In contrast, FSH levels were unaffected by insulin receptor deletion, but significantly decreased in IR/LepR^Kiss^ males (Fig. 6D). Overall, the additional deletion of leptin receptors restored LH levels, suppressed FSH levels and tended to decrease the age of puberty in males.

In females, vaginal opening was delayed by nearly 3 days in IR^Kiss^ mice, while IR/LepR^Kiss^ females exhibited an intermediate phenotype (Fig. 6B). The first day of estrus was significantly delayed in the double knockout mice, while IR^Kiss^ females exhibited an intermediate phenotype between the controls and IR/LepR^Kiss^ mice (Fig. 6B). Similar to the males, IR^Kiss^ females showed a significant reduction in LH levels that was restored in the double knockout animals Fig. 6C). While FSH levels were unaffected by deletion of insulin receptors from *Kiss1* neurons, deletion of both IRs and LepRs suppressed FSH levels (Fig. 6D). Overall, the additional deletion of leptin receptors restored LH levels, suppressed FSH levels and tended to decrease the age of puberty in females.

### Adult fertility of IR/LepR^Kiss^ mice

**Fig 7:**
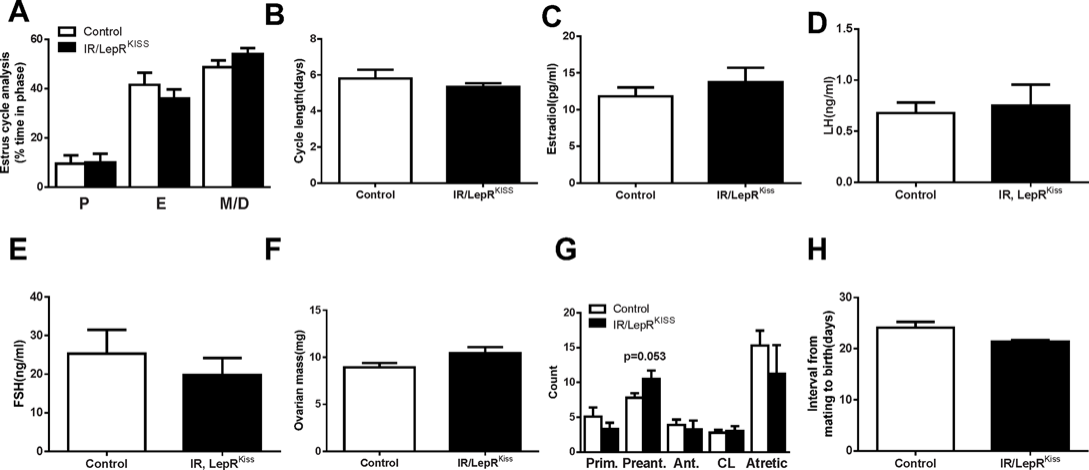
Female reproduction in IR/LepR^Kiss^ mice. (A) Estrous cycle analysis (Control, n = 6; IR/LepR^Kiss^, n = 5) and (B) estrous cycle length (Control, n = 6; IR/LepR^Kiss^, n = 5) in 3 months old females. (C) Estradiol in adult females in control (n = 9) and IR/LepR^Kiss^ (n = 6) mice. (D-E) LH and FSH levels in adult female control (n = 10) and IR/LepR^Kiss^ (n = 16) mice. (F) Ovarian mass in adult females in control (n = 9) and IR/LepR^Kiss^ (n = 6) mice. (G) Representative light photomicrographs of an adult IR/LepR^Kiss^ mouse ovary (n = 4). CL, corpora lutea. Scale bar, 100μm. (H) Fertility data from 5–6 months old females paired with established male breeders. Interval from mating to birth of a litter and litter size were compared between control (n = 12) and IR/LepR^Kiss^ (n = 6) mice.* *P*<0.05.

Adult female IR/LepR^Kiss^ mice showed normal estrous cyclicity, characterized by normal cycle length and progression (Fig. 7A and 7B). Female knockout mice also showed no difference in circulating levels of estradiol, LH, and FSH on diestrus (Fig. 7C-E). Female IR/LepR^Kiss^ mice had similar ovarian weights (Fig. 7F). Despite a trend toward increased preantral follicles in the ovaries of IR/LepR^Kiss^ mice, a comparable number of corpora lutea and follicles at all stages of maturation were found in control and IR/LepR^Kiss^ females (Fig. 7G). For analysis of fertility, mice were paired with established wild-type male breeders. The latency to birth was comparable between the two groups (Fig. 7H). Thus, fertility appears to be normal in these animals.

**Fig 8:**
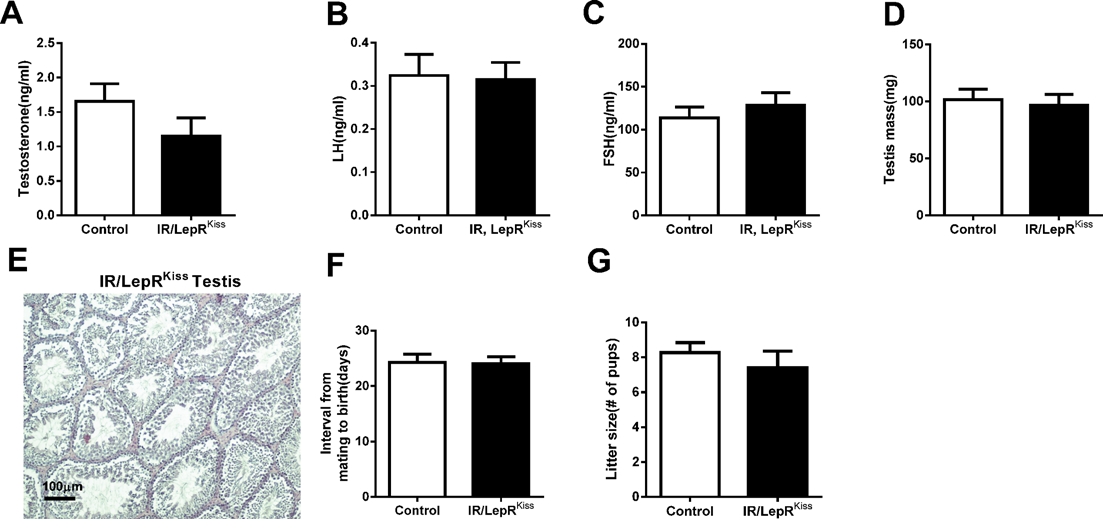
Male reproduction in IR/LepR^Kiss^ mice. (A) Testosterone in adult males in control (n = 9) and IR/LepR^Kiss^ (n = 8) mice. (B-C) LH and FSH levels in adult male control (n = 10–12) and IR/LepR^Kiss^ (n = 10) mice. (D) Testis mass in control (n = 9) and IR/LepR^Kiss^ (n = 8) mice. (E) Representative sections of adult testis in IR/LepR^Kiss^ mice (n = 4). Scale bar, 100μm. (F-G) Fertility data from 5–6 months old males paired with established female breeders. Interval from mating to birth of a litter and litter size were compared between control (n = 12) and IR/LepR^Kiss^ (n = 11) mice.

Male IR/LepR^Kiss^ mice showed no significant difference in levels of testosterone, LH or FSH in adulthood (Fig. 8A-C). Furthermore, testis weight was similar to controls in adult IR/LepR^Kiss^ mice (Fig. 8D). Testis histology showed all stages of spermatogenesis in seminiferous tubules and interstitial Leydig cells with normal morphology in IR/LepR^Kiss^ mice (Fig. 8E). Fertility, characterized by litter size and days required to impregnate a female, was not different between control and IR/LepR^Kiss^ males (Fig. 8F-G).

### Metabolic status of IR/LepR^Kiss^ mice

**Fig 9:**
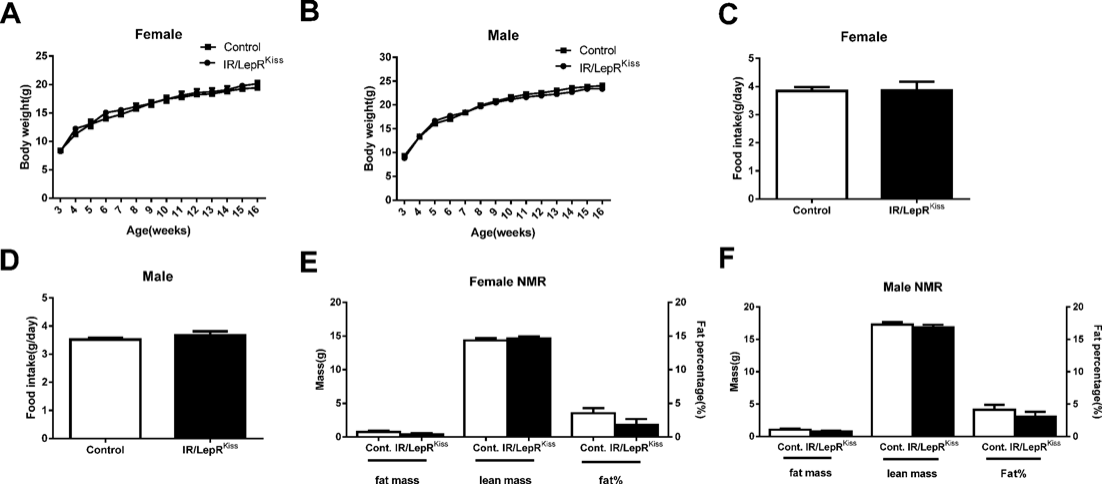
Metabolic phenotype in IR/LepR^Kiss^ mice. (A) Weekly body weight of female mice (Control n = 15 and IR/LepR^Kiss^ n = 8) mice (B) Weekly body weight of male mice (n = 16 each group). (C) Female average daily food intake calculated from weekly measurement. (D) Male average daily food intake calculated from weekly measurement. (E) Female body composition at the age of 4 months in control (n = 13) and IR/LepR^Kiss^ (n = 7) mice. (F) Male body composition at the age of 4 months in control (n = 15) and IR/LepR^Kiss^ (n = 12) mice.

Due to the potential interaction between *Kiss1* neurons and metabolic circuits in the hypothalamus [67], we examined the metabolic phenotype of IR/LepR^Kiss^ mice. The body weight of both females and males showed no significant difference between control and knockout mice (Fig. 9A and 9B). Likewise, average daily food intake was comparable between mice 2–3 months old of both sexes (Fig. 9C and 9D). Fat mass, lean mass and fat percentages were comparable in both females and males (Fig. 9E and 9F). Leptin levels were also similar between the two groups (Table 5).

**Table 5:** Overnight fast serum insulin and leptin.

	Sex	Control	IR/LepR^Kiss^
Serum insulin	Female	300.6 ± 42.5 n = 13	287.8 ± 60.7 n = 8
(pg/ml)	Male	258 ± 38.3 n = 16	399.8.0 ± 49.7 n = 7 * p = 0.0458
Serum leptin	Female	2.6 ± 1.1 n = 9	1.4 ± 0.7 n = 8
(ng/ml)	Male	1.4 ± 0.3 n = 11	1.3 ± 0.2 n = 9

**Fig 10:**
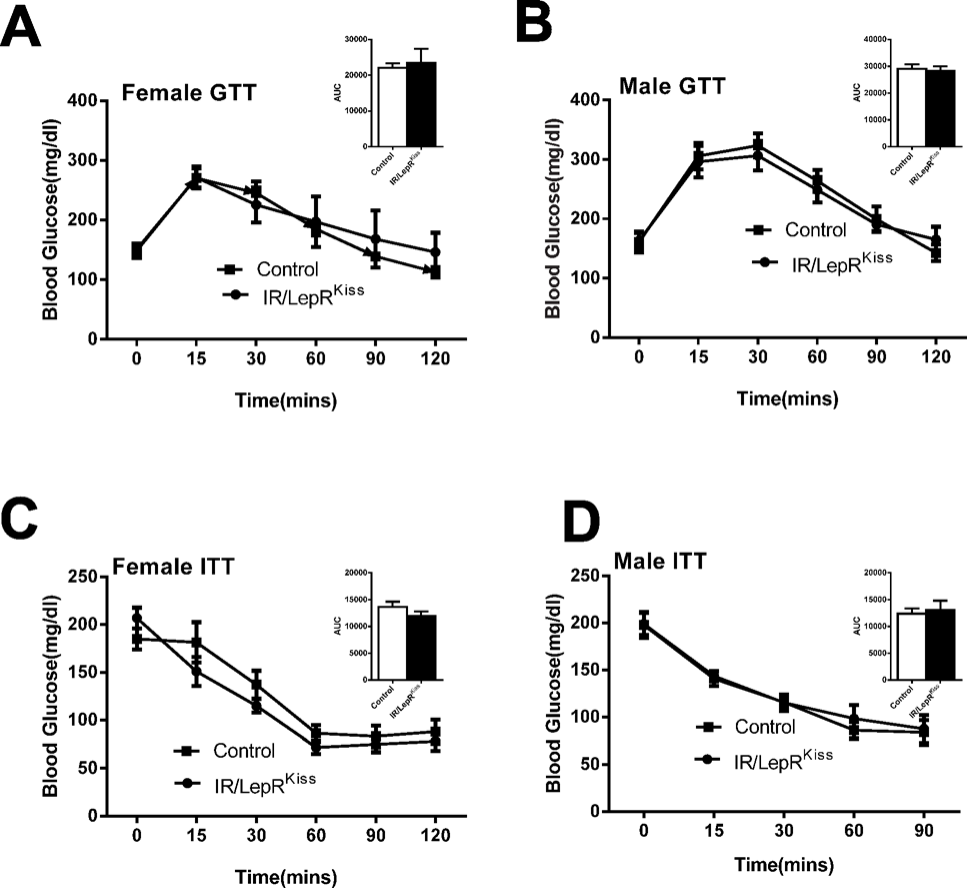
Glucose regulation in IR/LepR^Kiss^ mice (A) 4–5 months old female GTT and area under curve (AUC) (inset) in control (n = 11) and IR/LepR^Kiss^ (n = 5) mice. (B) 4–5 months old male GTT and AUC (inset) in control (n = 10) and IR/LepR^Kiss^ (n = 8) mice. (C) 5–6 months old female ITT and AUC (inset) in control and IR/LepR^Kiss^ mice (n = 7). (D) 5–6 months old male ITT and AUC (inset) in control (n = 11) and IR/LepR^Kiss^ (n = 7) mice.

Since *Kiss1* expressing cells in the hypothalamus, liver, or pancreas have the potential to influence glucose homeostasis, we examined relevant markers of glucose disregulation in IR/LepR^Kiss^ mice. We found no change in fasting glucose levels because of LepR deletion from *Kiss1* neurons (Fig. 10A and 10B). Overnight fasting serum insulin was unchanged in female IR/LepR^Kiss^ mice, but slightly higher in males (Table 5). Finally, glucose tolerance and insulin tolerance were normal in both sexes of IR/LepR^Kiss^ mice (Fig. 10A-10D).

## Discussion

Insulin appears to play a role in the early phases of puberty. The number of kisspeptin neurons in the AVPV in females increases sevenfold across puberty [68]. Insulin appears to contribute to this increase [42]. This action may involve the PI3K pathway since female mice lacking p110 isoforms in kisspeptin neurons also show lower numbers of Kiss1 neurons in the AVPV. These changes are likely to promote the establishment of normal preovulatory surges in females.

Recently, Evans and colleagues reported that only 5% of kisspeptin-immunoreactive cells in the hypothalamus showed co-labeling with IR-β [43]. This percentage is substantially lower than the 22% co-localization of IR mRNA and kisspeptin immunoreactivity we previously reported in the ARC [42]. Further, they found that a 200mU subcutaneous injection of porcine insulin to C57BL/6J mice did not cause Akt phosphorylation in *Kiss1* neurons. We therefore sought further evidence of *Kiss1* responsiveness to insulin. Using whole-cell recordings in the AVPV and ARC, we found that while the majority of *Kiss1* neurons were unresponsive to insulin, roughly a quarter (26.6%) of cells hyperpolarized in response to a 200nM concentration of insulin. Evans and coworkers also found that high concentrations of insulin (10U, i.p.) activated all AVPV *Kiss1* neurons and 17% of ARC neurons in an IR independent manner, possibly through IGF receptors. We believe that the electrophysiological responses we have observed are specific, because the concentrations used were physiological and in line with previously used concentrations inducing IR activation [69, 70].

Insulin has been shown to hyperpolarize several hypothalamic cell types, including leptin-inhibited and leptin-excited neurons, through activation of K_ATP_ channels [69–72]. Our results demonstrate that a subpopulation of *Kiss1* neurons in the AVPV and ARC are similarly hyperpolarized in response to insulin. Recently, Qiu and coworkers reported that guinea pig *Kiss1* neurons and pro-opiomelanocortin (POMC) neurons are depolarized by guinea pig insulin application [73]. It should be noted that guinea pig insulin is unique; of 51 residues, the A and B chains of guinea pig insulin differ from human insulin at 18 positions while most other mammalian insulins differ from each other at only 1–3 sites [74, 75]. In addition, *Kiss1* neuronal populations of guinea pigs and other rodents exhibit significant functional differences [76, 77], so insulin may act differently on *Kiss1* neurons in this species. Qiu and colleagues also suggested that zinc contamination of insulin preparations leads to hyperpolarization of POMC neurons in mice. This assertion was supported by experiments demonstrating hyperpolarization of mouse POMC neurons in response to zinc-containing Humulin and Novolin but not “pure” bovine or human recombinant insulin from Sigma-Aldrich. However, the zinc content of the Sigma-Aldrich formulations they used is 0.4–0.7% dry basis (Sigma-Aldrich, personal communication), comparable to the zinc content of Humulin. Using pharmacological and genetic techniques, we and others have demonstrated that insulin hyperpolarization of POMC neurons is dependent on the IR and its signaling pathways [70–72, 78]. In these studies, we therefore attribute the hyperpolarization of *Kiss1* neurons in response to insulin to a direct effect of the peptide.

Obesity involves multiple metabolic changes, including hyperleptinemia and hyperinsulinemia, which may contribute to the impaired fertility seen in the obese state. High-fat diet feeding, commonly used to induce obesity and hyperinsulinemia in animal models, is known to impair fertility. For instance, high fat feeding reduces testosterone, LH, and *Kiss1* levels and inhibits LH responses to kisspeptin in male rats [79]. Others have shown that insulin signaling in the pituitary gonadotroph or ovarian theca cell contributes to infertility in female DIO mice [80, 81]. We therefore tested the possibility that insulin signaling in *Kiss1* neurons impacts LH secretion and fertility in the obese state. Our findings argue against insulin playing a role in the hypogonadism associated with obesity. The reproductive phenotype of IR^Kiss^ mice on a high fat diet matched that of wild-type littermates, who were equally obese and hyperinsulinemic.

Chronic subnutrition has been shown to arrest puberty and reduce *Kiss1* mRNA levels in the arcuate nucleus (ARC) in female rats [82]. Repeated central injections of kisspeptin to these rats can restore pubertal progression despite the persistent caloric restriction [83]. In support of the idea that low insulin levels may serve as one of the signals of such chronic subnutrition, IR ablation from *Kiss1* neurons caused a moderate delay of puberty onset during the pubertal transition ([42], current study). Our current findings suggest that the effects of insulin on pubertal timing are modified by the actions of leptin. While IR ablation reduced LH levels mid-puberty and left FSH levels unaffected, the additional deletion of LepRs restored LH levels and suppressed FSH.

Numerous reports have provided evidence that leptin is a significant upregulator of hypothalamic *Kiss1* expression [25, 83–88]. Although one study demonstrated that up to 40% of *Kiss1* neurons in the ARC of male mice appear to have functional LepRs [85], subsequent studies challenged this finding by contending that LepRs were rarely colocalized with *Kiss1* cell [59, 89]. We found no leptin receptor expression in Kiss1 neurons in female mice on days P21–25 (several days prior to any sign of puberty) [41]. On P60–70, however, 8–15% of ARC *Kiss1* neurons coexpressed the LepR, depending on estrogen levels [41, 48]. However, there was no examination of when leptin receptor expression arose between these two time points. It is reasonable to think this occurred at some point during puberty. We suggest that leptin receptor expression is released from repression in the kisspeptin neuron at the initiation of puberty, as are other genes [90], allowing it to alter Kiss1 function as puberty progresses.

Single deletion and reexpression studies clearly showed that puberty can proceed without leptin signaling in Kiss1 neurons and demonstrated conclusively that mice lacking leptin fail to develop sexually due to leptin actions elsewhere in the brain [41, 59]. In other words, any effects of leptin in Kiss1 neurons are not sufficient to induce puberty. The current results augment these findings by showing that leptin plays a nonessential role in modifying the timing of puberty and interacts with the actions of insulin.

It is unclear whether these effects are due to the activation of intracellular signaling pathways that alter gene expression or due to direct actions on the excitability of Kiss1 neurons. If the former, it is plausible that leptin inhibits transcription of genes that promote GnRH release (or vice versa) specifically in Kiss1 neurons, although no evidence for a candidate gene has yet been identified (e.g. [59, 91]). Such an action could occur via STAT3 mediated gene transcription and be opposed by insulin signaling. Alternatively, the opposing effects of insulin and leptin on Kiss1 neuronal excitability may underlie their contrasting actions on pubertal timing. We have previously shown that leptin raises the membrane potential and increases the activity of a subset of kisspeptin neurons[41]. In contrast, the current study shows that insulin hyperpolarizes Kiss1 neurons. In both the case of leptin and insulin, their actions on Kiss1 neuron excitability have little functional relevance in adulthood, as they do not affect adult reproductive function; nevertheless, Kiss1 neurons may be particularly sensitive to these effects during the pubertal transition.

Our original assumption was that depolarization should increase kisspeptin release and hyperpolarization decrease it. However, recently an interesting alternative interpretation has been proposed [92]. While single-action potential-generated calcium influx is ideal for the release of classical neurotransmitters, burst firing promotes the release of neuropeptides [93–95].The “pacemaker” current is required for rhythmic firing, and virtually all Kiss1 neurons in both the ARC and RP3V express this current [96–98]. The pacemaker current can only depolarizes neurons from hyperpolarized states [99, 100]. Thus, inhibitory synaptic inputs may be necessary for burst firing to occur. We have demonstrated that prepubertal Arc Kiss1 neurons exhibit higher inhibitory presynaptic activity, but an action potential frequency significantly higher compared to that of Arc Kiss1 neurons recorded from adult females. Therefore, it is possible that insulin-induced hyperpolarlization promotes burst-firing while leptin inhibits it. However, it is unclear why, in this scenario, leptin receptor deletion alone did not advance puberty by increasing Kiss1 firing rates.

These finding raise the interesting possibility that these classic metabolic factors influence the frequency of GnRH pulsatile release during puberty by altering *Kiss1* neuronal activity. The frequency and amplitude of GnRH pulses varies developmentally and determines the relative proportions of LH and FSH synthesis and secretion [101, 102]. Increased frequency of pulsatile hypothalamic GnRH release favors LHβ gene transcription over FSHβ and increases the ratio of secreted LH to FSH, while a decreased GnRH pulse frequency favors FSHβ [101, 103–106]. The idea that leptin and insulin alter GnRH pulse frequency via actions on *Kiss1* neurons during puberty remains to be tested. Despite this influence on LH and FSH secretion, neither metabolic factor is essential for puberty. Indeed, genetic factors may mask the role of insulin sensing by *Kiss1* neurons during the pubertal transition [43]. In its absence, compensatory mechanisms complete the maturation of the reproductive axis. The *Kiss1* neuron may sense other circulating metabolic factors with important roles in this regard [107–109]. Indeed, ghrelin appears to modulate arcuate *Kiss1* activity in an estradiol dependent manner [108]. In addition, the *Kiss1* neuron may play a key role in transmitting metabolic information from upstream neurons, such as those that release NPY, α-MSH, and melanin-concentrating hormone. These functions remain to be elucidated.

*Kiss1* neurons synapse onto neurons such as POMC neurons that powerfully regulate glucose homeostasis and energy expenditure [88, 110, 111]. Indeed, kisspeptin receptor knockout females exhibit obesity, reduced energy expenditure and impaired glucose tolerance [112]. However, IR/LepR^Kiss^ mice exhibited normal weight gain, body composition, and glucose and insulin tolerance. Thus, deletion of *Kiss1* LepR and IRs did not alter any influence that *Kiss1* neurons have on melanocortin and other neuronal circuits controlling glucose and energy regulation. *Kiss1* is also reportedly expressed in several peripheral tissues, including the liver and α and β cells of the pancreas [60, 113–116]. Pancreatic α-cell targeted disruption of insulin receptor expression in mice results in glucose intolerance and hyperglycemia [117], not seen in these animals. Specific deletion of the β cell insulin receptor causes a loss of insulin secretion in response to glucose and fasting hyperinsulinemia and impaired glucose tolerance by 6 months of age. Given that we saw an isolated increase in fasting serum insulin levels in males, we conclude that if β cells expressing *Kiss1* and cre recombinase exist in this line, their number is too small to greatly impact glucose homeostasis.

In summary, in the presence of genetic or obesity-induced concurrent insulin and leptin resistance, *Kiss1* neurons are able to maintain reproductive function. In contrast, LepR and IR deletion in *Kiss1* neurons altered pubertal timing as well as LH and FSH levels in mid-puberty in a reciprocal manner. Our results confirm that *Kiss1* neurons do not directly mediate the critical role that insulin and leptin play in reproduction. However, kisspeptin neurons may experience a critical window of susceptibility to the influence of metabolic factors during puberty that can modify the onset of fertility.
